# Cardiac magnetic resonance adenosine perfusion at 3 Tesla is superior to 1.5 Tesla for detection of relevant coronary artery stenosis

**DOI:** 10.1186/1532-429X-13-S1-P97

**Published:** 2011-02-02

**Authors:** Peter Bernhardt, Robert Gradinger, Thomas Walcher, Wolfgang Rottbauer

**Affiliations:** 1 University of Ulm, Ulm, Germany

## Background

Cardiac magnetic resonance imaging (CMR) including adenosine perfusion and late gadolinium enhancement (LGE) at 1.5 Tesla (T) has been established for noninvasive detection of relevant coronary artery disease (CAD). However, little is known about the potential advantages of 3.0-T to detect CAD. The aim of our prospective study was to compare a compiled clinical routine CMR protocol performed at both 1.5-T and 3.0-T in patients with suspected CAD undergoing coronary x-ray angiography.

## Methods

Fifty-two patients (62.3 ± 10.2 years) with suspected CAD referred for coronary x-ray angiography were included into the study. All patients were scanned at both 1.5-T and 3.0-T including functional imaging, adenosine stress and rest perfusion, and LGE imaging. CMR images were analyzed by two blinded readers in consensus. A significant CAD was diagnosed by quantitative coronary analysis. Two thresholds of >50% and >70% stenosis in coronary arteries with a diameter of >2mm were chosen.

## Results

Diagnostic accuracy of the combined analysis of perfusion and LGE imaging yielded better values at 1.5-T and 3.0-T than the analysis of perfusion images alone. Sensitivity and specificity at 3.0-T was superior to 1.5-T in detection of coronary stenoses >50% (0.87 vs. 0.77 and 0.91 vs. 0.76) and >70% (0.96 vs. 0.89 and 0.92 vs. 0.80). Area under receiver-operator characteristic (ROC) curve was higher for detection of coronary stenoses in the left anterior descending and left circumflex artery at 3.0-T in comparison to 1.5-T, but not in the right coronary artery.

## Conclusions

This study showed that CMR at 3.0-T in a routine clinical setting is superior to 1.5-T in detection of significant CAD. 3.0-T might become the preferred CMR field strength for evaluation of CAD in clinical practice.

